# Relevance of Saliva Analyses in Terms of Etiological Factors, Biomarkers, and Indicators of Disease Course in Patients with Multiple Sclerosis—A Review

**DOI:** 10.3390/ijms252312559

**Published:** 2024-11-22

**Authors:** Aleksandra Kapel-Reguła, Irena Duś-Ilnicka, Małgorzata Radwan-Oczko

**Affiliations:** 1 Private Dental Practice AL-DENTA, 48-303 Nysa, Poland; ola_kapel@o2.pl; 2 Oral Pathology Department, Faculty of Dentistry, Wroclaw Medical University, 50-425 Wrocław, Poland; malgorzata.radwan-oczko@umw.edu.pl

**Keywords:** multiple sclerosis, saliva, blood, biomaterial

## Abstract

Multiple sclerosis (MS) is a demyelinating, progressive, and neurodegenerative disease. The cause of this condition remains unknown. Diagnosing and monitoring the course of this disease requires the use of time-consuming, costly, and invasive methods such as magnetic resonance imaging and cerebrospinal fluid analysis. To date, no specific diagnostic tests for MS are available. The purpose of this publication is to answer the question of whether saliva, as a mirror of oral and general health and easily obtainable test material, can be a significant source of information on etiological factors, biomarkers, and indicators of disease progression and whether analysis of substances in saliva is sensitive enough to replace plasma, urine, or cerebrospinal fluid. For this purpose, a systematic search of databases was conducted: PubMed, Google Scholar, and Embase.

## 1. Introduction

Multiple sclerosis (MS) is a chronic demyelinating disease associated with multifocal damage to the central nervous system (CNS). It affects the white matter as well as the gray matter, but gray matter pathology appears to play a decisive role in the development of physical and cognitive disabilities in affected individuals. A morphological reflection of the pathological process in the CNS is demyelination plaques or foci of damage to the myelin sheath of axons. Demyelination plaques are oval-shaped, ranging in size from a few millimeters to a few centimeters. They occur around small venous vessels, mainly in the periventricular region, in the corpus callosum, cerebellum, and cervical segment of the spinal cord [1,2,3]. The incidence of MS is increasing worldwide [2].

**Figure 1:**
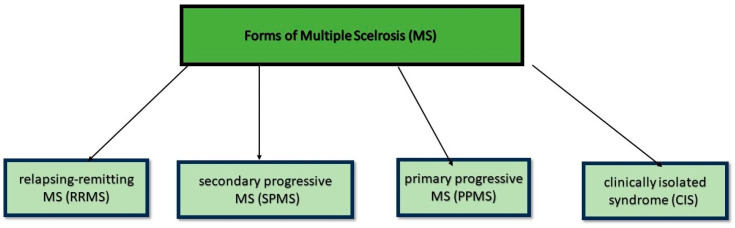
Differences between the forms of the Multiple Sclerosis.

MS is the most common non-traumatic disabling disease that affects young adults, and its clinical representation is presented in Figure 1.

It is widely accepted that autoimmune diseases such as MS result from complex interactions between individual genetic susceptibility and environmental factors. Infectious and non-infectious environmental factors present in childhood and young adulthood have been identified as strong determinants of MS risk. Viral, bacterial, and fungal microbial infections can act as triggers to induce autoimmunity [3]. There are two prevailing theories to explain the autoimmune etiology of MS. The first posits that autoreactive CD4+ T cells are activated peripherally and cross the blood–brain barrier to reach the CNS. Once in the CNS, CD4+ T cells are reactivated by local antigen-presenting cells, triggering an inflammatory response that induces the recruitment of other leukocytes (such as T cells, B cells, and macrophages). The second hypothesis suggests that MS is primarily a neurodegenerative disease that elicits an autoimmune response. The migration of peripheral leukocytes across the blood–brain barrier is a critical step in initiating relapses. Infiltration of pro-inflammatory leukocytes into the CNS leads to further disruption of the myelin sheath, ultimately causing neuronal loss. This hypothesis has been corroborated in mouse models of MS, particularly experimental autoimmune encephalomyelitis (EAE). Therefore, a deeper understanding of the factors stimulating peripheral leukocyte infiltration into the CNS and the mechanism of inflammation in MS offers the potential to identify initiating factors, biomarkers, and disease progression indicators [4,5].

To confirm the diagnosis of MS and monitor the course of the disease, magnetic resonance imaging (MRI) is used to visualize lesions and to study oligoclonal IgG groups within the cerebrospinal fluid (CSF). MRI is a time-consuming and expensive technique, while analysis of CSF obtained by lumbar puncture is a highly invasive procedure [6]. To date, no specific diagnostic tests for MS are available [7,8]. Hence, there is a need for research geared toward the discovery of new markers and indicators that, in conjunction with existing clinical symptoms, can be used for a more precise initial diagnosis, monitoring of the condition, and observing the effectiveness of treatment.

The potential use of saliva as a body fluid in the treatment of oral and systemic diseases has been widely studied. As a mirror of oral and general health, saliva has been shown to provide valuable information. It contains not only proteins secreted by salivary glands but also proteins derived from gingival fluid, oral microflora, and plasma proteins transported from blood to saliva via cellular pathways [9].

The composition of saliva is a consequence of the exocrine contribution of three pairs of major salivary glands, a variable number of smaller salivary glands, as well as various non-exocrine components, such as exfoliated oral epithelial cells, leukocytes, microorganisms, and serum-like fluid exuded from the epithelial mucosa and gingival (crevicular) fluid. Due to the contribution of mucosal and gingival fluid, substances transported into the circulatory system are also present in saliva [10].

Apart from water, less than 1% of saliva contains mucins, i.e., humectant proteins, proline-rich glycoproteins, and components of the innate immune system. The typical salivary protein concentrations are 0.7–2.4 mg/mL, although there is wide variability depending on the time of collection, sex, age, and pathological conditions. Many of the proteins undergo post-translational modifications through glycosylation, acetylation, phosphorylation, and proteolysis. Saliva also contains hormones and growth factors [9].

The regulation of salivary flow and its secretory functions is directly controlled by the parasympathetic nervous system, specifically the facial nerve (VII) and the glossopharyngeal nerve (IX). This link between saliva and the nervous system suggests that certain proteins may provide information about neurological disorders [11], and specific markers associated with neurodegenerative diseases can be detected in saliva. Studies have shown that individuals with neurodegenerative diseases have significantly altered levels of pathogenic proteins, including Aβ, tau, α-syn, and HTT, as well as those involved in inflammation and oxidative stress [12].

The saliva proteome has 30% in common with blood plasma. Saliva analysis is gaining popularity. This is due to the easy availability, non-invasive, stress-free way of obtaining the material and the reproducibility of the samples tested [13]. Personnel collecting saliva do not require specialized training, and the risk of infection is minimal. It is also worth noting the economic aspect, as collection and storage of the material requires only basic equipment [9]. However, it is important to state in this part of this review that saliva tests for MS biomarkers are not part of clinical procedures and are still under the horizontal line from a scientific perspective.

Since saliva contains so many different substances and its acquisition is non-invasive, risk-free, and inexpensive, one has to wonder what the role and relevance of saliva analysis are in the diagnosis of MS. The purpose of this systematic review was to answer the question of whether saliva is an important source of information about a patient with MS. Can the substances in it be treated as biomarkers of disease initiation and progression? And is the analysis of substances in saliva sensitive enough to replace plasma, urine, or CSF? This systematic review aimed to assess diverse salivary parameters to be implemented to provide insight into the possible diagnostic methods available or to be explored in the future. Saliva, as an easily accessible biomaterial, might represent a valuable use for MS patients, and as such, the evaluation of those parameters might help with future diagnosis and prognostics of the disease.

**Figure 2:**
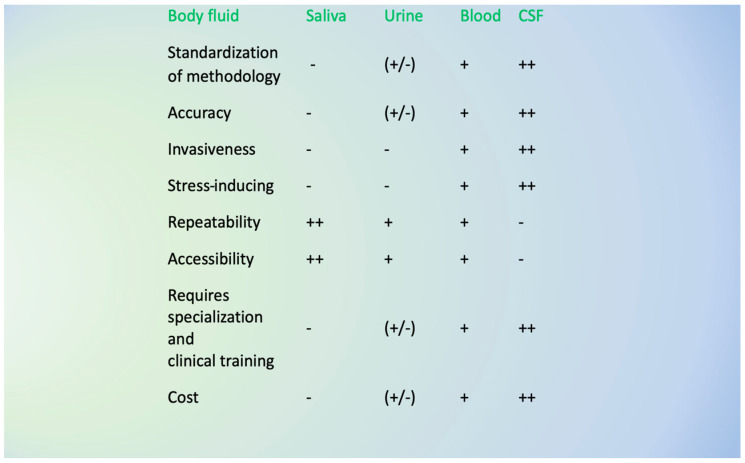
Advantages and disadvantages of biological fluid analysis in MS. Legend: (++) very high (+) high (+/-) medium (-) low.

The advantages and disadvantages of biological fluids used to detect clinical biomarkers are presented in Figure 2 below.

## 2. Methods

### 2.1. Search for Articles

This systematic review was based on the results of a database search from June to September 2023. The Preferred Reporting Items for Systematic Review and Meta-Analysis (PRISMA) guidelines were followed. Articles were selected from the electronic databases PubMed, Google Scholar, and Embase. Keywords used were [multiple sclerosis AND saliva] and [multiple sclerosis AND saliva AND research].

### 2.2. Article Selection Criteria

Articles selected for this review were evaluated independently by two evaluators and met the following inclusion criteria: (1) the sample consisted of patients with MS (aged 18 and older) who had their biomaterial analyzed; (2) articles were published between 2000 and 2023; (3) the study group of patients had MS, with no diseases of other etiologies; (4) the full text of the article was available in the database; and (5) the entire article was in English.

### 2.3. Exclusion Criteria

Publications that were excluded were review articles, book chapters, and abstracts published in journals.

### 2.4. Data Acquisition

In the initial evaluation, only abstracts of the articles were read and reviewed by A.K.-R. and I.D-I. If the data were not sufficient, the evaluators read the Methods and Results of the studies. Publications were accepted if they met all criteria and were read and comprehensively analyzed.

**Figure 3:**
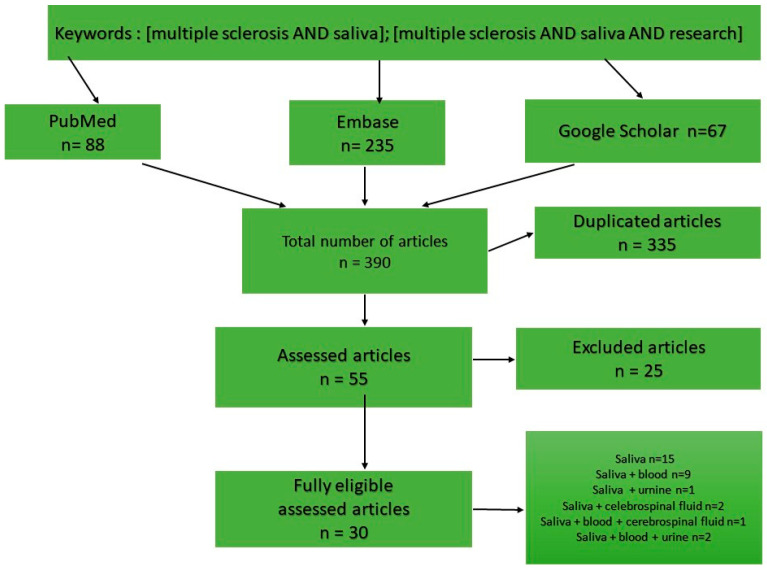
PRISMA checklist for this manuscript’s methodology of obtaining the saliva of patients with MS.

The analysis was performed qualitatively, considering the type of assay in saliva. Results of the studies were placed in a table with the following information: authors and year of publication, type of assay, objectives of the study, sample size, and significance of the assay. The findings of the present systematic review were reported following the Preferred Reporting Items for the PRISMA checklist in Figure 3.

## 3. Results and Discussion 

### 3.1. Microbiological Tests

Ten articles were selected that addressed the topic of microbiological studies of saliva in patients with multiple sclerosis: eight involved viruses, one bacteria, and one fungi.

#### 3.1.1. Virological Testing of the Saliva of SM Patients

The publications selected for this systematic review undertook studies on Epstein–Bar virus (EBV) [14,15,16,17], human herpes virus type 6 (HHV 6) [18,19,20], and cytomegalovirus (CMV) [21], as well as the determination of the presence of HHV 6 and EBV in the saliva of MS patients [15].

Researchers’ interest in EBV stems from the fact that it is transmitted through oral secretions and is a B-lymphotropic herpesvirus. Once the virus enters the oral cavity and throat through saliva, it infects epithelial cells and salivary glands. EBV also infects B cells, which contain the specific CD21 receptor (CR2) for the virus on their surface. The virus causes cell lysis and multiplies at a rapid rate. The proliferation of EBV-infected B cells causes enlargement of the lymphoid tissue of the lymph nodes and palatine tonsils. Memory B cells circulate in the blood, spreading throughout the body and serving as a reservoir—the infection progresses to a latent form that most likely persists throughout life [16]. There are indications that EBV infections are a strong risk factor for MS. Primary EBV infection (infectious mononucleosis) increases the risk of MS and occurs before the clinical manifestations of the disease [14,15,16,17].

Similarly, HHV 6 is considered a potential infectious agent associated with MS pathogenesis due to its neurotropic effects. Primary infections can cause neurological complications. They are characterized by latency and periodic reactivation [20].

CMV belongs to the Herpesviridae. Viral infections develop between 10 and 35 years of age and are asymptomatic. However, in patients with autoimmune diseases, high titers of anti-CMV antibodies indicative of acute infection have been found in laboratory tests. The virus can be found in most tissues and organs as well as body fluids, especially in urine and saliva during the active phase of infections [21].

Accordingly, publications have investigated the impact of the presence, activity, and reactivation of individual viruses in conjunction with disease activity [14,15,16,17], humoral response [21], and viral activity following valacyclovir treatment [15].

Methods for the isolation of EBV DNA [14,15,16,17]; CMV [21]; and HHV 6 [18,19,20] were used.

Holden and co-authors [17] analyzed salivary EBV levels in three cohorts of MS patients. The results showed that EBV lytic activity in a patient could not be inferred from a single measurement of EBV in saliva. In addition, the subjects did not consistently behave as “EBV disseminators” or “EBV non-disseminators”.

Latham et al. [14] evaluated the correlation between EBV and HHV 6 viral immune activity determined by blood and saliva testing and MRI lesion activity. The results showed that EBV and HHV 6 DNA in saliva was detected significantly more often than in peripheral blood mononuclear cells (PBMCs). As for the correlation between the amount of DNA in saliva and the activity of MRI lesions, no significant relationship was observed. The association between the immune activity of the virus and the activity of MRI lesions was confirmed based on the patient’s blood. Saliva is a good material for detecting viral DNA [14]. Similar observations were confirmed by Höllsberg et al. [15]. Their analysis assessed the presence of EBV and HHV-6 DNA in patient saliva and plasma during a randomized, double-blind trial of valacyclovir. Patients with MS had EBV and HHV-6B DNA in both saliva and plasma, but only EBV expression in saliva was significantly reduced after valacyclovir treatment. Although EBV and HHV-6B DNA can be detected in plasma in healthy individuals, co-expression of both viruses in MS patients was highly significant and associated with clinical activity.

Sangol et al. [21] assessed the prevalence of CMV in patients with various subtypes of MS. Saliva, serum, plasma, and PBMCs were screened for anti-CMV antibodies and CMV-DNA. A significantly higher prevalence of CMV-DNA in saliva, serum, and urine was observed in patients compared to controls. In addition, systemic CMV infections were found in 25.5% of patients and only 3.2% of controls.

Ramroodi et al. [20] monitored active HHV 6 infections in Iranian patients with different subtypes of MS. It is noteworthy that the ratio between MS patients and healthy volunteers carrying (presenting) the virus DNA in saliva and serum was at a similar level. Viral DNA was detected in all saliva samples that had previously shown the presence of viral DNA in PBMCs, both in patients and controls. Discrepancies in the frequency of virus detection within the respective bodily fluids were observed. Most frequently, DNA HHV6 was detected in blood serum (in 60% of MS patients) and less often in CSF (in 30% of SM sufferers), while only 11.5% of cases revealed the presence of the virus in saliva.

The study by Gieß et al. [16] seems to contradict the claim that saliva is a good material for assessing the presence of EBV. The study evaluated the relationship between radiological and clinical disease activity and EBV antibodies in serum and DNA in saliva. It was estimated that the amount of EBV DNA in saliva did not differ between the patient and control groups. The authors also did not confirm an association between the level of viral antibodies and the amount of virus in saliva and radiological or clinical disease activity. Similarly, Akhyani et al. [18] evaluated the distribution and characteristics of HHV 6 variants. Virus distribution was studied in saliva, peripheral blood lymphocytes (PBLs), serum, and urine. Analysis of the urine and serum of patients and controls showed the presence of the virus only in patients with MS. In studies of PBLs and saliva, there were no statistical differences in the prevalence of HHV 6 between patients with MS and healthy subjects.

Summing up, seven articles analyzing the saliva of MS patients for viral infections were reviewed. In five articles [14,15,17,20,21], saliva was recognized as an appropriate and reliable material for the determination of levels of both EBV and HHV 6. In one study [14], EBV and HHV 6 DNA were more frequently detected in saliva than in PBMCs. In two studies [16,18], the viral content in the saliva of healthy and diseased subjects showed no significant differences, in contrast to the significant viral levels of the urine and serum tests of patients and controls.

#### 3.1.2. Bacterial Testing of the Saliva of SM Patients

Bacteria and other microorganisms have significant effects on the nervous system and thus play a role in neurological diseases. In MS, bacterial infection leads to an increase in T helper lymphocytes and induces the production of pro-inflammatory cytokines (interleukins: IL-21, IL-17, and IL-22).

In a study by Zangeneh et al. [22], saliva samples and oral swabs were collected from 30 patients and 30 healthy volunteers to compare the diversity of bacterial populations in the oral cavity.

In MS patients, the levels of Staphylococcus, Fusobacterium, Bacteroides, Porphyromonas, Prevotella, Veillonella, Actinomyces, Propionibacterium, and Bifidobacterium were higher, and of Peptostreptococcus, Micrococcus, Enterococcus, and Lactobacillus were lower.

The number of bacteria detected by each method was significantly higher in the patient group, which may support the premise that oral microorganisms can alleviate or exacerbate inflammation that affects the pathogenesis of MS. It can be hypothesized that the control of oral infections may result in a reduction in the progression of MS.

Analysis of saliva by DGGE electrophoresis can detect a greater number of bacterial genera or species compared to culture methods, as evidenced by the fact that at least 10 different bacteria were isolated in each sample, while the number of bacteria isolated by culture methods was much lower.

#### 3.1.3. Mycological Testing of the Saliva of MS Patients

da Cunha et al. [23] evaluated the prevalence of *Candida* spp. in the oral cavity of MS patients relative to a control group, based on the premise that polymorphonuclear cells from MS patients, regardless of whether immunosuppressive therapy was used or not, reduced in vitro phagocytic activity against pathogens such as *Candida albicans*.

*Candida* species can be found in the oral cavity as commensal microorganisms and can become pathogenic in the presence of predisposing factors such as immunosuppression.

The project involved 100 subjects, aged between 18 and 68 years: 55 patients diagnosed with MS according to the McDonald (2017) criteria and 45 healthy individuals [23]. Saliva samples were collected and inoculated on *Candida*-selective culture media. After a 48 h incubation period, colony-forming units (CFU/mL) were counted. Results were analyzed. The analysis showed that the colonization of *Candida* spp. in the oral cavity of MS patients was higher than in the control group; however, the results were not proven to be statistically significant. Regarding the specification of *Candida* species, it is worth noting that *C. tropicalis* and *C. krusei* were found only in the MS patient group.

Saliva, therefore, is a good and readily available medium for conducting mycological analyses. As shown in the study by da Cunha et al. [23], patients with MS had higher levels of *Candida* colonization in the mouth relative to the control group (despite a statistically significant difference) and were characterized by specific fungal species.

### 3.2. Examination of Inorganic Constituents in Saliva

Fluctuations in electrolytes within body fluids can be linked to neurological and immunological disorders characteristic of MS [24]. The analysis of the inorganic components of saliva was performed in two publications [24,25] selected for this review. The electrolytes studied were calcium, assayed in both works [24,25], potassium [24], and phosphorus [25].

The studies included 84 [24] to 25 [25] MS patients and healthy subjects as a control group.

Salivary calcium was studied due to the premise of an increased incidence of MS in areas with less soil calcium content [24]. In some countries, it is common practice to prescribe cholecalciferol in high doses to patients with MS because of its potential for immunomodulation and reducing relapse rates. Cholecalciferol increases serum calcium levels, and there appears to be an additive effect in patients still taking calcium supplements. Elevated salivary calcium levels may be associated with increased bone mass loss and lower bone mineral density. In turn, bone mass loss may cause calcium to be released into the blood and then into the saliva [25]. The variety of processes occurring in both the external and internal environment seems to confirm the results obtained. In the study by [24], salivary calcium concentrations in MS patients were statistically significantly lower than in healthy subjects. The opposite result was obtained by Mortazavi et al. [25]. In that study, salivary calcium levels were significantly higher in MS patients than in controls. This difference may be related to various patient characteristics, such as duration of illness, place of residence, type of medications and supplements taken, or size of the study group and calcium detection methods.

Potassium in the salivary glands is responsible for fluid secretion and osmotic pressure regulation. Higher amounts of potassium, in saliva can cause a decrease in nerve excitability. Due to damage to the myelin sheath in MS, there is often a slowing down of impulse stimulation in the optic nerve, brain, and spinal cord. Hence, the higher level of potassium in the saliva of sclerosis patients obtained in the study by [24] compared to a control group of healthy people.

In a study by Mortazavi et al. [25], salivary phosphorus levels were significantly higher in MS patients than in controls, and this may be due to the fact that the daily intake of phosphorus, potassium, manganese, and copper in Iranian MS patients was higher than the standard recommended intake.

Although the results for calcium determination were discrepant, statistically significant differences were obtained between patients and controls for each of the elements determined in the study. Saliva, therefore, appears to be a good medium for determining electrolyte levels. However, considering the limited number of available publications, further studies are necessary to capture differences in electrolyte composition between sick and healthy subjects and to try to answer the question of what causes this condition.

### 3.3. Hormone and Enzyme Tests

Eight papers qualified for this review—five of them [26,27,28,29,30] dealt with cortisol in the saliva of MS patients, two described differences in melatonin (MT) levels [31,32], and one focused on the analysis of acetylcholinesterase [33].

### 3.4. Cortisol

Researchers’ interest in cortisol stems from the fact that it is a key regulator of the immune system, energy metabolism [34], and stress. There are several mechanisms by which this adrenal product and participant in the hypothalamic–pituitary–adrenal (HPA) axis, may be relevant to fatigue in MS [35]. Studies support a positive association between stressful experiences and the risk of exacerbating MS symptoms [36,37]. Hypocortisolemia has been frequently observed in chronic fatigue syndrome [27]. Previous studies have shown that the HPA axis is overactive in MS patients. In many analyses, cortisol levels are elevated in patients with MS, regardless of the body fluid in which cortisol levels were assessed [38].

Each of the papers [26,27,28,29,30] selected for this systematic review examined unstimulated cortisol secretion activity in MS patients and healthy control subjects.

Analyses were designed to determine diurnal changes in cortisol in association with fatigue [26,27], yawning [29], and depressive symptoms [27,28], and in the Gold et al. study [30], the correlation with changes in hippocampal subregional volume determined by MRI was examined in addition to mood disorders.

In each study, cortisol levels were determined using patients’ saliva. Due to the specificity of the analyses, the material was collected in different ways. Thompson et al. [29] collected saliva before and after stimuli simulating yawning. Hildebrandt et al. [26] collected samples at 8 a.m., before and after performing a task lasting about 25 min and requiring divided attention. This procedure was repeated at 4 p.m. In studies [27,28,30], participants independently, after appropriate training, collected saliva six times per day (four times in the morning and at 3 p.m. and 10 p.m.) [28], three times (after waking, at 4 p.m., and at 9 p.m.) [30], and nine times per day [27]. Significantly, in all studies [26,27,28,29,39], cortisol levels determined in the saliva of patients always differed from the control group.

Diurnal cortisol changes in people with MS fluctuated. Patients showed elevated diurnal cortisol levels during the first hour after waking (CAR) [26,27,28]. A difference in cortisol levels in the afternoon was observed in a study by Hildebrandt et al. [26], while Kern et al. [28] and Powell et al. [27] reported no such change. Gold et al. [30] showed subtle changes in cortisol profiles with slightly elevated evening levels in patients with MS but unchanged morning levels, although the results were not statistically significant.

Kern et al. [28] found that patients with relapsing–remitting MS and moderately elevated depression scores, as determined by the Beck Depression Inventory (BDI), showed significantly higher cortisol levels in the CAR and statistically different cortisol release compared to healthy volunteers. Similarly, in the study by Gold et al. [30], patients with MS and depressive symptoms showed higher cortisol levels and smaller hippocampal volumes. The opposite results were obtained by Powell et al. [27], where elevated CAR values were obtained in subjects with RRMS who did not have major depressive disorder.

In a study evaluating the association of diurnal cortisol secretion with fatigue [26,27], patients with relapsing–remitting MS were found to have elevated cortisol levels upon awakening. Interestingly, in a study by Hilberbrandt et al. [26], who divided MS patients into those suffering from fatigue and those without fatigue (MS-NF), the group that did not complain of cognitive fatigue showed elevated cortisol levels in the morning and afternoon. The researchers suggested that MS-NF patients suffer from dysregulation of diurnal cortisol levels. However, this claim is contradicted in the study by Powell et al. [27], in which no significant statistical differences were observed between patients who reported fatigue and those who did not report these complaints.

In a study by Thompson et al. [29], which determined the correlation between cortisol levels and the yawning reflex, it was found that there was a significant difference between healthy and sick subjects who did not yawn, as opposed to those who were sick but yawned. These results support the hypothesis that cortisol levels increase during yawning. The cortisol levels of participants with MS (who did not yawn) were significantly different from those of healthy participants. It is also worth noting that not all participants who yawned showed results that were due to not reaching threshold cortisol levels compared to healthy participants who did not yawn. These findings support Thompson’s cortisol hypothesis [29], which states that yawning occurs after threshold cortisol levels are reached to lower brain temperature.

Cortisol levels did not correlate with disability [26,27,28], gender [26,27,28,29], the use of immunomodulatory drugs, or forms of MS [26,27,28].

Saliva thus appears to be a good medium when it comes to the importance of cortisol. In each of the studies, significant differences were noted between sick and healthy subjects. The inconclusiveness when it comes to linking salivary levels of the hormone to fatigue or depressive symptoms is due to the influence of many variables rather than the body fluid used in the analysis itself.

### 3.5. Melatonin

MT (N-acetyl-5-methoxytryptamine) is a natural hormone secreted by the pineal gland. The suprachiasmatic nucleus receives signals from the retina depending on the level of sunlight and sends feedback to the pineal gland, which regulates the release of MT. At lower sunlight levels, circulating levels of the hormone increase via local MT release to retinal ganglion cells and Th17 cells [40].

This hormone is interesting because it inhibits pro-inflammatory Th17 cells [41], which appear to play a key role in the pathogenesis of various autoimmune diseases, including MS [42]. MT also exerts anti-inflammatory effects by inhibiting pro-inflammatory cytokines such as IL-6 and INF-γ and promotes the release of IL-10 in the spleen and CNS [43]. MT levels have been linked to the severity and relapse rates of MS. It has been confirmed that MT can have beneficial effects on some of the MS symptoms, improving patients’ quality of life [44].

Two studies qualified for this review: [31,32]. One evaluated the role of MT in the pathogenesis of MS [32], while the other studied the diurnal release of MT in relapsing–remitting MS [31]. The studies included 35 [32] to 55 [31] MS patients matched with an appropriate control group. MT levels were determined in the patients’ saliva, with unstimulated saliva samples collected once in low light at 6 p.m. in the study by Ghorbani et al. [32], while in the study by [31], the subjects collected saliva samples themselves nine times a day at designated time points. Despite the differences in the sampling method and the different specificity of conducting the survey, both studies did not confirm the assumed thesis.

Ghorbani et al. [32] evaluated the role of MT in the pathogenesis of MS in conjunction with the involvement of ultraviolet light from sunlight. No significant difference was found between MT levels in the saliva of patients and healthy subjects. However, taking into account the effect of age, older patients were found to have significantly lower MT levels than the control group. There was no difference in MT levels between groups with good or poor sleep quality. Mean MT levels were higher in men than women but without statistical significance. The level of the hormone did not correlate with the first attack, type of treatment, or degree of disability. Similarly, the study by [31] found no evidence of a generally disturbed circadian rhythm of MT in patients with relapsing–remitting MS. MT levels correlated only moderately with fatigue.

Based on the analyses of the two studies, some discrepancies were noted. Ghorbani et al. [32] found no correlation between MT levels and disease duration, while Kern et al. [31] observed that longer disease duration was associated with significantly lower MT levels. As for the degree of disability, one paper [32] found no correlation with hormone levels, while the other study [31] found a moderate association.

### 3.6. Acetylcholinesterase

Most MS patients experience inflammation. Studies have shown that inflammation stimulates the vagus nerve and releases acetylcholine (ACH) from its end [45]. ACH can inhibit the production of pro-inflammatory cytokines and directly suppress inflammation [46]. The amount of ACH depends on the balance between its production and its degradation by hydrolysis. There are reports of reduced acetylcholinesterase activity in various chronic and severe inflammatory diseases [47].

Thirty women with MS and 30 healthy female volunteers constituting the control group were enrolled in the study by [33]. Acetylcholinesterase activity was measured by a photometric method in serum and stimulated and unstimulated saliva.

In the MS group, the mean acetylcholinesterase activity in serum, unstimulated, and stimulated saliva was significantly lower than in the control group. It is worth noting that the researchers observed a positive correlation of acetylcholinesterase activity between serum and unstimulated and stimulated saliva. Therefore, it can be concluded that acetylcholinesterase activity in serum and saliva may have diagnostic value. However, given the simplicity and non-invasiveness of saliva sampling, measuring activity by this means seems to be a better option.

### 3.7. Biomarkers for MS Diagnostic Purposes

Despite the many studies on MS, there is no single diagnostic test to make a diagnosis monitor the progression of the disease, or evaluate the effectiveness of pharmacotherapy. Therefore, research is underway to discover an effective biomarker, that is, a characteristic substance that could be objectively measured and evaluated as an indicator of the disease process or pharmacological response to therapeutic intervention [48]. Despite many studies aimed at identifying potential markers, no marker has been validated for MS. Hence, there is a need to find a substance that can confirm the diagnosis and monitor disease progression, response to treatment, and prognosis in MS [49].

In 12 articles selected for review, seven different substances were evaluated as potential biomarkers: myelin basic protein (MBP) [50], tau protein [51], immunoglobulin light chains [52,53], human HLA compatibility antigens [54,55], immunoglobulin 1 IIβ [56], and oxidative stress markers [57,58], and one publication [6] analyzed 119 saliva peptides to find differences in protein levels between MS patients and healthy individuals.

#### 3.7.1. Saliva Profile in MS Patients

Due to its content of proteins from various sources, i.e., salivary glands [59], gingival fluid [60], oral microflora, and plasma [61], human saliva reflects not only the condition of the oral cavity but also of the whole body. Various systemic disorders have been shown to quantitatively and qualitatively affect the saliva proteome [62,63,64]. In a study by Manconi et al. [6], a quantitative analysis of 119 peptides/saliva proteins was performed to find qualitative and/or quantitative differences in saliva proteins in patients with MS compared to healthy volunteers. The study was designed to determine potential biomarkers. The study group consisted of 49 individuals (of both sexes) with diagnosed MS receiving various drug therapies (32 individuals) and treatment-naïve patients (17 individuals). Fifty-four healthy volunteers (men and women) constituted a demographically and ethnically matched control group. Resting whole saliva samples were collected with a soft plastic aspirator at the base of the tongue between 9 a.m. and 1 p.m. Protein testing was performed by mass spectroscopy. Statistical analysis showed different levels of 23 proteins. Eight proteins showed lower levels in MS patients compared to controls, and these were mono- and di-oxygenated cystatin SN, mono- and di-oxygenated cystatin S1, mono-oxygenated cystatin SA, and mono-phosphorylated statherin. Fifteen proteins showed higher levels in MS patients relative to the control group, and these were antileukoproteinase, two proteoforms of prolactin-induced protein, PC peptide (Fr. 1–14, Fr. 26–44, and Fr. 36–44), SV1 fragment of statherin, SN cystatin Des1–4, SN cystatin P11 variant L, and cystatin A T96. The differences observed by the researchers were mainly due to different levels of proteins involved in inflammatory processes or the immune response triggered by the condition. However, some of the differences may have been related to the side effects of therapies used in MS. Therefore, further analyses are needed to determine the changes in proteins in different forms of MS and also to determine the dependence of the concentration of specific peptides on the degree of disability.

#### 3.7.2. Myelin Basic Protein

MBP is the second most common protein in the CNS after proteolipid protein (PLP) and consists of 30% of total protein and about 10% of myelin dry weight. It is the only structural protein so far found to be essential for myelin formation in the CNS and has been called the “myelin execution molecule” [65]. MBP is being investigated as a factor in the autoimmune pathogenesis of MS. MS is characterized by inflammation of the nervous system, demyelination, and axonal loss. One of the main theories of MS pathogenesis suggests that exposure to foreign antigens causes activation of inter-reactive T cells in genetically susceptible individuals. MBP is a possible autoantigen [66]. Although the direct role of MBP as a primary antigen in MS has yet to be definitively confirmed, the study of changes in MBP levels may serve as an indicator of the disease.

In the study by Mirzaii-Dizgah et al. [50], MBP levels were determined in the serum and stimulated and unstimulated saliva. The study was conducted on 29 healthy women and 32 patients with relapsing–remitting MS. MBP levels were determined using an enzyme-linked immunosorbent assay (ELISA) kit (Bioassay Technology Laboratory, Shanghai, China). 

MBP levels in stimulated saliva were significantly lower in MS patients than in the healthy group. Similarly, the serum protein concentrations were lower in female patients. However, the difference in MBP concentrations in unstimulated saliva between women with MS and healthy women was not significant.

Thus, the use of MBP as a potential marker in saliva for diagnosing MS seems promising. It is worth noting, however, that only one study was found for this review. Thus, it is necessary to increase the study group, expand demographic and ethnic diversity, and study patients with other forms of MS.

#### 3.7.3. TAU Protein

Tau is a major microtubule-associated protein (MAP) that forms the scaffolding of the neuronal cytoskeleton and supports cellular transport. However, due to specific disorders, tau protein can become neurotoxic by affecting deficient oxidative phosphorylation and apoptotic activity, which causes mitochondrial fragmentation, leading to neurodegeneration. As a result of certain disorders, tau protein can be translocated in the cell body and dendrites of nerve cells. As it translocates, it becomes aggregated and blocks axons, leading to degeneration of neurons [67]. After nerve cell damage, these proteins are released into the extracellular space [68].

A publication qualified for this review examined the levels of total tau protein in serum and stimulated and unstimulated saliva [51]. The study included 30 healthy women and 30 patients with MS. Venous blood and saliva were collected from each participant at the same time in the morning. Tau protein levels were determined by ELISA. Mean total serum tau protein levels were lower in the MS patients than in the healthy group. Statistical analysis showed no significant differences in total tau protein concentrations in both stimulated and unstimulated saliva between healthy and diseased women. There was also no significant correlation between total tau protein in saliva and disability scores (EDSS). The results showed that salivary tau protein does not seem to be a good marker for diagnosing MS. However, it is worth noting that only one study was found on this subject, which involved a rather small group, limited to females. It would be necessary to conduct further studies on a larger and more diverse group to determine the usefulness of tau protein determination in the saliva of MS patients.

#### 3.7.4. Immunoglobulin Light Chains (FLCs)

Intrathecal synthesis of immunoglobulins is commonly observed in diseases of the CNS of infectious or autoimmune origins. This process has been shown to be of great diagnostic value. Over the past decade, the intrathecal production of not only intact Ig immunoglobulins but also free Ig light chains (FLCs) has gained considerable interest in the diagnosis of MS. A growing body of evidence strongly suggests that FLC production is significantly elevated in MS and that quantification of FLCs in the CSF can contribute to diagnosis [69]. Kaplan et al. [52] and Lotan et al. [53], based on hypotheses of increased numbers of immunoreactive cells in saliva and tears, determined FLC titers in saliva of MS patients. The studies were aimed at developing and applying a new procedure for testing FLCs in the saliva of healthy individuals and patients with MS [52] and evaluating the usefulness of salivary immunoglobulin light chain determination as a biomarker of disease activity and response to treatment in MS [53].

The projects involved 85 patients with MS (73 with relapsing–remitting MS and 12 with a secondary progressive MS) and 28 healthy subjects as a control group [52]. Another study included 55 patients with MS and 40 healthy volunteers [53].

In both studies, the procedure was based on Western blot analysis to detect and semi-quantitatively evaluate monomeric and dimeric FLCs [52,53].

Saliva was collected from MS patients and healthy participants constituting the control group [52,53]. Saliva samples were examined by determining the total FLC levels, and an index was calculated to determine the monomer-to-dimer ratio of free immunoglobulin chains.

A study by Kaplan et al. [52], based on a statistically significant FLC index value, distinguished healthy individuals from MS patients and patients with active MS from those in remission. Analysis of monomeric/dimeric FLCs showed that most patients with active MS had a higher proportion of monomeric FLCs, and on this basis, it was possible to distinguish a healthy person from a patient with active MS with high sensitivity and specificity.

Lothan et al. [53], by analyzing FLC levels, distinguished those with active disease from those in stable remission. FLC levels were significantly higher for the active form of the disease characterized by MRI changes that were enhanced after gadolinium administration. Similarly, immunoglobulin light chain analysis distinguished patients on therapy. Patients in remission treated with disease-modifying therapies had significantly lower FLC levels compared to untreated patients.

The advantage of salivary FLC analysis [52] is that there is no effect of food intake on FLC determination. However, this method has some limitations, as no correlation was observed between the degree of neurological disability and changes in T2 [53] and FLC levels.

Immunoglobulin light chains can become a useful biomarker to distinguish between a healthy person and a patient with active MS. Determining the level of immunoglobulin light chains determines disease activity and response to treatment. It is noteworthy that the developed procedure is non-invasive, does not require expensive equipment, and may find application in clinical laboratories as a new tool to help diagnose and monitor MS.

#### 3.7.5. Cytokines

Researchers’ interest in the pro-inflammatory cytokine IL-1β stems from the fact that it is one of the main mediators of the disease. Subjective fatigue experienced by MS patients is linked to peripheral inflammation. This is a sickness behavior arising from cytokine-driven alterations in brain regions that process internal bodily sensations [70,71,72].

Hanken et al. [56] investigated the difference in the levels of pro-inflammatory cytokines in patients with relapsing–remitting and secondary progressive forms of MS. They evaluated IL-1β levels as an indicator of fatigue in patients with different clinical courses. The effect of disease-modifying drugs on peripheral inflammatory markers was also analyzed.

The study included 116 patients with MS (62 with the relapsing–remitting MS and 54 with the secondary progressive form) and 51 healthy controls. IL-1β levels in saliva were determined using an ELISA. Fatigue was assessed using various fatigue scales.

The analysis of results showed that IL-1β levels allowed for the assessment of fatigue in patients with relapsing–remitting MS. Patients with secondary progressive MS had elevated IL-1β levels compared to patients with relapsing–remitting MS and the group of healthy volunteers. Elevated cytokine levels in patients with secondary progressive MS may be attributed to their higher mean age and greater disability (EDSS). Reduced motor function diminishes immune system activity, consequently increasing IL-1β levels. Similarly, a general increase in systemic pro-inflammatory cytokines from this group was observed with advancing age [73]. No significant differences in IL-1β levels were observed between relapsing–remitting MS patients and healthy subjects. Disease-modifying therapy had a significant effect on IL-1β levels, as treated patients showed lower IL-1β levels compared to untreated patients.

Determining IL-1ß levels can be helpful in differentiating between the secondary progressive and relapsing–remitting forms of MS and assessing the effect of pharmacotherapy.

#### 3.7.6. HLA Diagnosis from Patient Saliva

Major human leukocyte antigens (HLAs) are bound to cells but are present in trace amounts as soluble forms circulating in serum, plasma, and other human body fluids [74]. Soluble HLA class I (sHLA-I) and class II (sHLA-II) particles may have an immunomodulatory function [75]. In healthy individuals, serum levels of sHLA-I and sHLA-II are stable [74]. However, serum levels of sHLA-I are significantly elevated in patients with various rheumatic, autoimmune and inflammatory diseases [76,77]. sHLA-I is typically found in very low amounts in saliva, sweat, urine, and/or tears of healthy individuals, while sHLA-II is routinely detected in all body fluids [74] except serum [78]. The potential role of soluble sHLA in the pathogenesis of MS has not been sufficiently investigated. It can be expected that the measurement of sHLA in CSF will most likely reflect CNS disease activity and act as a biological marker of response to immunomodulatory treatment in MS.

In terms of HLA determination in saliva, two studies were eligible for review [54,55]. Adamshivli [54] aimed to answer the question of whether the measurement of soluble HLA in body fluids (saliva and CSF) can play a role in assessing autoimmune disease activity. Minagar et al. [55] studied sHLA class II molecules in the saliva of patients with MS as a potential marker of therapeutic responses to high-dose interferon beta-1a.

The study group consisted of 17 Caucasian patients with projection–remitting MS [55] and 13 controls who were also Caucasian [54]. The study group was selected based on ancestry, as there are indications that racial–ethnic factors may also affect sHLA levels [54]. Human tissue compatibility antigen levels were determined by ELISA.

In both studies included in this systematic review [54,55], the mean levels of sHLA-II in the saliva of patients with MS were significantly higher than in the control group. The measurement of sHLA-I was below diagnostic sensitivity and, therefore, had no diagnostic value [55]. Adamashvilli et al. [54] additionally noted that the mean level of sHLA-II in CSF was equivalent to the mean level of sHLA-II in saliva. No differences in sHLA-II levels in saliva and CSF were observed between MS patients with and without contrast-enhancing lesions on MRI, so antigen levels did not correlate with disease activity [55]. In a study by Minagar et al. [55] that determined salivary soluble HLA levels as a potential marker of response to interferon-β1 treatment, it was reported that sHLA-II values in saliva, before and after IFN β-1a treatment, showed a steady increase in mean concentrations. The increase in salivary sHLA-II values was associated with a stable clinical course and a decrease in the number of contrast-enhancing lesions on brain MRI.

Measuring the level of soluble class II HLAs can distinguish patients with MS from healthy people. It is noteworthy that saliva, in this case, can replace the difficult-to-perform and expensive analysis of CSF. Interestingly, serial measurement of salivary sHLA-II may serve as a potential therapeutic marker for the response to IFN β-1a treatment. HLA-II seems to be an interesting biomarker for future analysis, as there is a rather limited number of publications on this issue, in addition to a small and strictly ethnically selected study group. Further studies seem necessary to confirm the usefulness of HLA as a potential marker of the therapeutic efficacy of other MS drugs on larger and more diverse ethnic groups.

#### 3.7.7. Oxidative Stress Parameters from Saliva

Oxidative stress is a condition in which there is an imbalance between the excessive production of reactive oxygen species (ROS) and nitrogen and a relative deficiency of antioxidants. The CNS, due to its high content of polyunsaturated fatty acids that are prone to oxidation and high oxygen demand, is susceptible to free radicals, low concentrations of antioxidants, and antioxidant enzymes. Increased ROS leads to loss of integrity of the blood–brain barrier, destruction of myelin, and degeneration of nerve tissue. Therefore, oxidative stress is an important factor in the pathogenesis of many diseases, including neurodegenerative and neuroinflammatory diseases such as MS [79,80].

The subject of salivary oxidative stress was addressed by two articles that qualified for this review. Varol et al. [58] compared the levels of substances associated with neurodegeneration and inflammation, i.e., myeloperoxidase (MPO) and lactoferrin (LF), total antioxidant capacity (TAOC), and oxidative status (TOS), in the saliva of MS patients with healthy subjects in the context of periodontal health. They also studied the correlation between salivary oxidative status and systemic inflammation as determined by the neutrophil-to-lymphocyte ratio (NLR) determined in blood. The work by Karlik et al. [57] aimed to compare markers of oxidative stress in MS patients with healthy subjects. For this purpose, they measured the carbonyl stress and antioxidant status of saliva and serum.

The study group consisted of MS patients who had not used corticosteroids for 1 to 3 months, ranging from 29 [57] to 92 [58], while the control group consisted of sex- and age-matched healthy subjects. Blood samples from the ulnar vein and unstimulated saliva were collected once [58] and three times [57] (on the first day after admission to the hospital, after a dose of intravenous methylprednisolone therapy, and after 2–3 months of disease).

The colorimetric method determined TOS and TAOC levels and MPO and LF levels were determined by ELISA [58]. Advanced oxidation protein products (AOPPs) were determined by spectrophotometry. Lipoperoxidation markers were defined as thiobarbituric acid reactive substances (TBARSs) and quantified based on a calibration curve performed with 1,1,3,3-tetramethoxypropane. Advanced glycation end products (AGEs) and fructosamine were measured to evaluate carbonyl stress in the samples. As a marker of antioxidant status, saliva and plasma iron-reducing ability (FRAS/FRAP) was measured using the Benzie method [57].

In a study by Karlik et al. [57], higher levels of lipoperoxidation and carbonyl stress markers were noted in the plasma and saliva samples of MS patients, with the difference between patients and healthy subjects being more significant in saliva. There were discrepancies in protein oxidation markers (AOPPs), as serum AOPP levels were higher in people with MS, while no differences were noted in saliva between the two groups. An interesting discrepancy was observed in the case of TAOC. TAOC was lower in the patients’ serum, while the level was higher in saliva. For markers of antioxidant stress (FRAS), saliva seems to be the appropriate medium for determination, as their levels were significantly lower in MS patients, while in the plasma, there were no differences between healthy subjects and those with MS. The therapy used did not significantly alter the levels of oxidative stress markers in saliva or plasma. The main limitation of the presented study is the variability in the clinical condition and the different treatment of patients.

In the study by Varol et al. [58], patients with MS had a significant decrease in TAOC, higher levels of TOS and oxidative stress index, lower levels of MPO, and higher levels of LF compared to controls, but the differences were not statistically significant. A time dependency was observed, as patients with longer illnesses had higher levels of TAOC and MPO. Periodontal findings in patients with MS, except for a lower percentage of bleeding on probing (BOP%) in patients with relapsing–remitting MS, did not differ from the control group. However, by distinguishing the form of the disease, it was noted that patients with progressive MS had worse oral health, higher values of periodontal indices in terms of probing depth (PD), clinical attachment level (CAL), the gingival index (GI), and lower salivary flow rates (SFRs). Increased MPO and decreased TAOC in saliva, as well as higher NLR values in patients with MS, indicate a clear, ongoing systemic inflammation despite altered immune surveillance due to medications. Logistic regression analysis was performed to determine the periodontal parameters influencing MS and showed that the effects of TAOC, TOS, and NLR were statistically significant in determining the probability of MS in participants. TAOC values were found to have a negative effect on MS, while TOS and NLR had a positive effect.

Examination of lipoperoxidation and carbonyl stress markers [57] in saliva could become a potential differentiation tool, as significantly higher levels of these substances have been reported in MS patients than in healthy individuals. The same goes for markers of antioxidant stress (FRAS). However, in this case, the relationship was reversed, as lower levels of FRAS were reported in the saliva of patients than in healthy individuals. Markers of protein oxidation (AOPP) and TAC [57] need further study, as discrepancies between saliva and serum were observed in the study. The determination of TAOC and TOS may be a marker indicating the duration of the disease, and examination of oral health indicators may help distinguish between forms of MS. Determination of TAOC, TOS, and NLR levels may be a potential indicator of the onset of the condition.

## 4. Conclusions

**Figure 4:**
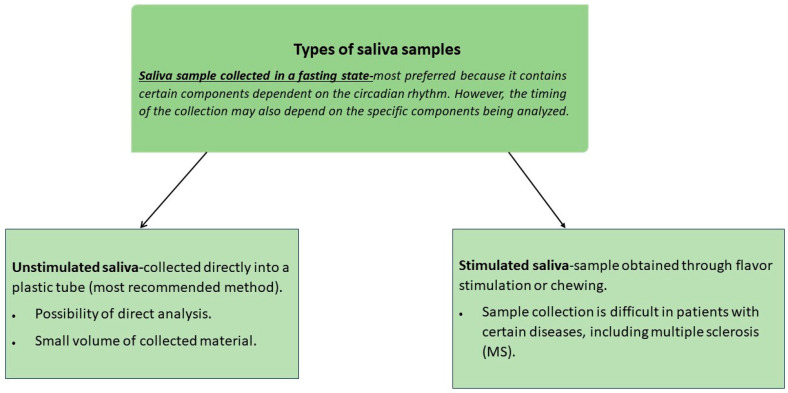
Types of saliva samples.

In this review, we selected 30 articles that analyzed stand-alone saliva samples and compared parameters in saliva with serum, urine, and CSF. In 16 articles: [6,22,23,24,25,26,27,28,29,30,32,39,52,53,55,56] the levels of various parameters in the saliva were evaluated. In each publication, saliva samples were collected from MS patients and healthy volunteers, constituting a control group. In 15 articles, unstimulated saliva was collected, while in one [22] examining the bacterial profile of the oral cavity, a swab was also taken. In nine publications [6,22,23,24,25,32,52,53,56], samples were collected once, while in five papers, [26,27,28,29,30] saliva was collected several times a day, and in two [39,55], it was collected at intervals of several months. In four studies [26,27,28,39], saliva was collected by participants at home. The relevance of the saliva for MS patients is shown in Figure 4.

It is noteworthy that only two of the fifteen articles analyzed found no significant statistical differences between healthy and diseased subjects. One of these studies examined MT levels [32] and one analyzed oral colonization by Candida fungi [23]. An interesting discrepancy occurred concerning calcium determination. In the study by Chałas et al. [24], calcium levels in the saliva of patients were statistically significantly lower than in the control group, while Mortazavi et al. [25] found statistically higher calcium levels in patients. Other publications, although they dealt with various issues, i.e., EBV [39], bacterial diversity [22], cortisol fluctuations [26,27,28,29,30], saliva protein profile [6], the role of individual substances as biomarkers [52,53,55,56] were found to have statistical significance determinations or were determined to be significantly different between patients and the control group.

The analysis of saliva and blood parameters was described in 12 articles (see Table 1) [14,15,16,18,19,20,21,33,50,51,57,58]. In 10 publications: [14,15,16,18,19,20,21,33,50,51], the same components were determined in saliva and blood. In two studies: [33,58], different parameters were chosen to determine inflammation [58] and disease activity [33] in saliva and serum. In papers [15,18,19,20,21,33,50,51,57,58], the saliva and blood of MS patients were compared with healthy volunteers. In the case of two publications [14,16], it was not necessary to include a control group due to the nature of the project. In each article eligible for review, serum was collected, and in five studies: [18,19,20,21,58], peripheral blood was also analyzed. In 10 publications, similar results were reported for parameter levels under study in the saliva and serum, both when statistically significant differences between patients and the control group were identified and when such discrepancies were not observed [16]. In a study by Karlik et al. [57] that analyzed oxidative stress markers, the same results were obtained in blood and saliva for hypoperoxidation and carbonyl stress markers, while a difference in levels was noted for AOPP, TAC, or FRAS in different media. The study by [18] examined the distribution of different herpes virus variants in the body and found no significant differences between the healthy group and patients in the case of saliva and peripheral blood, while a significant difference occurred in the case of serum analysis. It is also worth noting the differences between stimulated and unstimulated saliva and serum. This method of analysis was undertaken in three studies: [33,50,51]. In a publication examining acetylcholinesterase levels, both stimulated and unstimulated saliva and serum reported reduced acetylcholinesterase levels in MS patients. In a study on MBP [50], the same results, i.e., increased levels of MBP, were noted in serum and stimulated saliva (no difference in unstimulated saliva). In the case of tau protein in stimulated and unstimulated saliva, there were no significant differences between patients and controls. Differences between protein levels were observed for serum. The publication by [19] reported different results between serum and saliva. In the study [15] examining the distribution of EBV and HHV 6 in saliva and plasma, viruses were found in similar levels in both fluids in patients with MS. However, saliva appears to be the more sensitive medium for drugs, as after valacyclovir therapy, significantly decreased virus levels were found only in saliva. In the study on oxidative stress [58], saliva and peripheral blood samples were analyzed to determine inflammation. However, they were assessed by other parameters. MPO and LF levels were determined in saliva, while the NRL index was determined in blood. In this case, a discrepancy was noted; as the level of the NRL index indicates inflammation, this was not confirmed by LF or MPO parameters determined in saliva.

Three studies [18,21,31] included in this review examined the same parameters in saliva and urine in patients and healthy volunteers. In two of the publications [21,31], similar results were obtained in both body fluids. Kern et al. [31] found no evidence of a disturbed MT rhythm in MS patients, but higher levels of the hormone were observed in the patient’s saliva immediately after waking. A similar trend was noted in the nocturnal fraction of urine. In a study by Sanadgol et al. [21] analyzing the distribution of CMV in various body fluids, the authors found that the prevalence of CMV among MS patients in both urine and saliva was higher than in the control group and was further associated with an increase in IgG (saliva) and IgE (urine) antibodies. In the study [18] analyzing the distribution of HHV 6 in various body fluids, urine appeared to be a better medium, as a statistically higher prevalence of HHV 6 was noted in MS patients than in healthy subjects, while this pattern did not appear in saliva.

In two studies [20,54], the analysis of identical parameters in saliva and CSF in patients and healthy subjects was put together. It seems surprising that in both studies, the significance of the determination of the studied parameters in saliva and CSF was similar. In the study [54] evaluating the levels of soluble HLAs, it was found that the mean level of sHLA-II in CSF and saliva were equivalent and higher in the patient group, while sHLA-I levels were undetectable in saliva and CSF samples in patients with MS. Similarly, the study [20] analyzing the prevalence of HHV 6 in MS patients found that in saliva and CSF in relapsing–remitting and secondary progressive MS, the prevalence of HHV 6 virus in patients was at similar (but not statistically significant) levels. No viral DNA was detected in either fluid in any patient with primary–progressive MS. These data are included in the table summarizing publications on saliva with other media.

It is noteworthy that, considering the substances assayed in saliva in the various publications (some studies involved analysis of a clique of substances), of the 18 substances selected from the studies, 16 differed in levels in patients with MS versus healthy volunteers. One case [39] presented an intermediate result, i.e., a reduction in EBV titers in the saliva of patients after treatment with teriflunomide was obtained but without statistical significance. Two papers [23,32], failed to obtain significant differences in the studied parameters between patients and healthy subjects.

**Figure 5:**
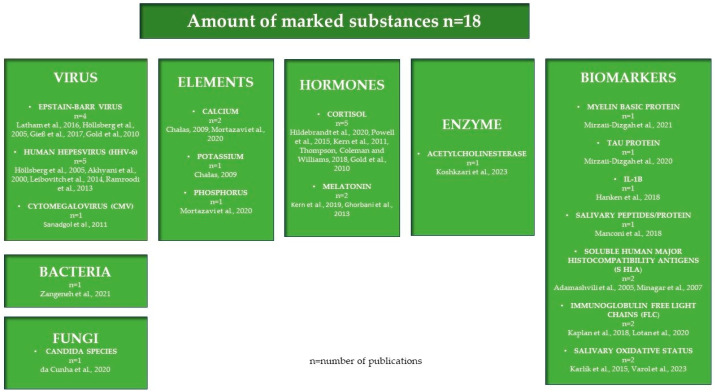
Substances analyzed with the use of biomarkers for the purpose of MS diagnostics [6,14,15,16,18,19,20,21,22,23,24,25,26,27,28,29,30,31,32,33,50,51,52,53,54,55,56,57,58].

In 20 publications analyzed in this review, 18 substances were determined, as shown in Figure 5.

As for the comparison of the same substances in saliva and in other media, i.e., blood (plasma, whole blood, and mononuclear cells), urine, and CSF, out of 16 determinations (some publications studied several different substances, and some studies determined the substance in several media), 11 cases [14,15,19,20,21,33,54] showed comparable results in saliva and another medium. It is noteworthy that for 3 determinations out of these 11, saliva seemed to be a better medium than blood, as comparable EBV DNA levels were observed in the saliva and plasma, but only in saliva did viral DNA decrease after the treatment of patients with valacyclovir [15]. Similarly, higher HHV 6 virus titers have been reported in the saliva of patients compared to blood [14,19]. In four assays comparing saliva with other media, an intermediate result was found. HHV 6B virus was present in the saliva and plasma of MS patients, but the presence of EBV and HHV 6 was strongly correlated only in the plasma of MS patients [51], while in a study by Mirzaii-Dizgah et al. [50], significant reductions in MBP levels were obtained in stimulated saliva and serum only. In unstimulated saliva, there was no correlation. Kern [31] obtained the same results in saliva and serum, but there was no evidence of a disturbed MT rhythm. In Karlik’s work analyzing five markers of oxidative stress, two bioindicators showed identical levels in saliva and plasma, while three showed discrepancies in the levels of individual media. In 1 of 17, the analysis of tau protein levels [51] showed lower levels of the substance in serum, while no differences were noted in the saliva of MS patients.

The cause of discrepancies in some results found in saliva and blood can be attributed to physiological variations in saliva composition, while the biochemical composition of blood is generally more stable. At the same time, the concentrations of biochemical compounds in circulation are well-documented, standardized, and defined within narrow reference ranges [81]. In the case of saliva, the lower stability of component concentrations poses a bioanalytical challenge, leading to a continued lack of standardization in analyses. It is also worth noting that many components in saliva can originate from plasma through passive diffusion or active transport, which presents significant diagnostic potential [82]. In any case, to reflect systemic bioactivity in a useful manner, a quantitative test for these parameters in saliva should be highly correlated with serum levels [83].

Due to the significant degree of similarity in the results with other media, it is worth emphasizing that biomarkers and other substances found in saliva seem to be promising diagnostic tools for monitoring the course of the disease and pharmacotherapy. Verification studies on existing substances appear to be essential, as well as attempts to discover new, easily identifiable, and unambiguous bioindicators. The potential applications of individual markers and other studied substances are presented in Table 2.

Considering the easy availability of the material, the non-invasive nature of sample collection, and the relatively low economic costs, saliva appears to be a noteworthy test material among patients with MS, which, in some cases, could replace less readily available media. At the same time, to establish saliva as an alternative matrix for other body fluids, it is necessary to precisely specify the reference values of compounds in saliva and to further develop bioanalytical technologies for saliva testing.

## Figures and Tables

**Table 1:** Biomaterial used in the analysis of MS-relevant biomarkers.

	Saliva + Blood	Saliva + Urine	Saliva + CSF
Number of publications	12	3	2
Convergent results (number of publications)	10	2	2

**Table 2:** Types of studies and provided analysis for the diverse parameters in MS diagnostics.

Study Name	Application
**Virological studies**	- The possibility of determining the levels of EBV and HHV 6 in patients with MS.
**Bacteriological studies**	- The difference in the levels of colonies of specific bacteria between individuals with MS and healthy volunteers. - Further analyses are necessary—small sample sizes and a limited number of publications.
**Inorganic ingredients**	- Higher levels of potassium and phosphorus in the saliva of people with MS (multiple sclerosis). - Ambiguous results regarding calcium levels. - Further analyses are necessary—limited number of publications, selected and small study groups.
**Cortisol**	- Differences in cortisol levels between patients with MS and healthy individuals. - Further research is necessary—inconclusive results in analyses comparing cortisol levels with additional parameters, e.g., fatigue and symptoms of depression.
**Melatonin**	- Further studies are necessary—results are inconsistent and ambiguous.
**Acetylcholinesterase**	- Higher activity of acetylcholinesterase in patients with MS. - Further analysis is necessary due to the limited number of publications and the selected and small study groups.
**Myelin basic protein (MBP)**	- Lower levels of MBP in stimulated saliva in women with MS. - Further analyses are necessary—limited number of publications, selected and small sample sizes.
**Tau protein**	- No differences in tau protein levels between sick and healthy individuals. - Further analyses are necessary to verify the obtained results due to the small number of studies and the limited sample size of the study groups.
**Light chain immunoglobulins (FLC)**	- A potential marker for distinguishing healthy individuals from patients with active forms of MS (multiple sclerosis). - The ability to assess the response to treatment. - Minimal equipment requirements. - Further studies are necessary—limited number of publications, small study groups.
**IL-1β cytokine**	- A potential marker distinguishing a patient with relapsing–remitting MS from a patient with secondary progressive MS. - The ability to assess the effectiveness of pharmacotherapy. - Further studies are necessary—limited number of publications, small study groups.
**Leukocyte antigens (HLA)**	- A potential marker for distinguishing a healthy person from a patient with MS. - The possibility of assessing the effectiveness of IFN β-1a therapy. - Further analyses are necessary due to the limited number of publications, ethnically selected samples, and the small size of the study groups.
**Parameters of oxidative stress**	- A potential marker distinguishing a healthy individual from a patient with MS (markers of lipid peroxidation, carbonyl stress, and antioxidant status (FRAS)). - The possibility of determining the duration of the disease (total oxidant capacity (TAOC), oxidant status (TOS)). - A potential indicator of disease onset (levels of TAOC and TOS). - Further analyses are necessary—limited number of publications.
